# Influence of Androgen Deprivation Therapy on the Development of Sarcopenia in Patients with Prostate Cancer: A Systematic Review

**DOI:** 10.3390/nu16050656

**Published:** 2024-02-26

**Authors:** Marta Stelmach-Mardas, Wojciech Warchoł, Aleksandra Garczyk, Emilia Warchoł, Jolanta Korczak, Maria Litwiniuk, Beata Brajer-Luftmann, Marcin Mardas

**Affiliations:** 1 Department of Obesity Treatment, Metabolic Disorders and Clinical Dietetics, Poznan University of Medical Sciences, 60-569 Poznan, Poland; garczykaleksandra@gmail.com (A.G.); emilaw@wp.pl (E.W.); 2 Department of Ophthalmology and Optometry, Poznan University of Medical Sciences, 60-806 Poznan, Poland; wwarchol@ump.edu.pl; 3 Department of Chemotherapy, The Greater Poland Cancer Center, 61-866 Poznan, Poland; jolanta.korczak@wco.pl; 4 Department of Cancer Pathology and Prevention, Poznan University of Medical Sciences, 61-866 Poznan, Poland; maria.litwiniuk@wco.pl; 5 Department of Pulmonology, Allergology and Pulmonary Oncology, Poznan University of Medical Sciences, 60-569 Poznan, Poland; bbrajer@ump.edu.pl; 6 Department of Gynecological Oncology, Institute of Oncology, Poznan University of Medical Sciences, 60-569 Poznan, Poland; marcin.mardas@ump.edu.pl

**Keywords:** androgen deprivation therapy, ADT, sarcopenia, prostate cancer, prostatic neoplasm, systematic review

## Abstract

The changes in body composition during androgen deprivation therapy (ADT) in patients suffering from prostate cancer (PCa) are recognized by professionals more often as biomarker for effective treatment. The aim of this study was to investigate the impact of ADT on the sarcopenia development in PCa. The following databases were used: PubMed, Embase, Web of Science and Scopus databases. Out of 2183 studies, 7 were included in this review. The fixed-effect model was used in the meta-analysis. A significant increase in SATI (Subcutaneous Adipose Tissue Index) of 0.32 (95% CI: 0.13–0.51) *p* = 0.001, decrease in SMI (Skeletal Muscle Index) of −0.38 (95% CI: −0.57 to −0.19) *p* < 0.0001, and SMD (Skeletal Muscle Density) of −0.46 (95% CI: −0.69 to −0.24) *p* < 0.0001 were observed. No statistical association was visible between ADT and changes in BMI (Body Mass Index), 0.05 (95% CI: −0.18–0.28), *p* = 0.686, and VATI (Visceral Adipose Tissue Index): 0.17 (95% CI: −0.02 to 0.37), *p* = 0.074. In conclusion, the ADT significantly contributes to the body composition changes and sarcopenia development.

## 1. Introduction

Prostate cancer (PCa) is one of the most frequent neoplasms in men, diagnosed mostly after the age of 60. More than 15% of men diagnosed with PCa present with disease that has spread beyond the prostate. Another 15% to 30% of men who undergo primary treatment will experience a return of their cancer. Androgen deprivation therapy (ADT: drugs to reduce the level of male hormones) has been the main treatment for advanced disease [1]. Body composition in prostate cancer (PCa) patients is of high importance as low muscle mass is associated with approximately 50% increased risk of mortality, where the total fat mass was not found to be a prognostic factor for survival. Specifically, higher subcutaneous fat and lower visceral-to-subcutaneous fat ratio are related to better patient survival [2,3]. It is also important to support the implementation of personalized physical activity (PA) in patients’ daily life as there is evidence suggesting that PA has beneficial effects on physical performance [4], lean body mass (LBM) and fat mass during androgen deprivation therapy (ADT) [5].

Low muscle mass is a relevant element in sarcopenia definition. Among the factors that make up the driving force in sarcopenia development are the following: aging, comorbidities, cancer, low physical activity and malnutrition [6]. Sarcopenia has been recognized as a disease since the 10th revision of the International Classification of Diseases (ICD-10) [7], which is supported by the newest ICD-11 [8]. According to the guidelines of the European Working Group on Sarcopenia in Older People (EWGSOP2), sarcopenia is considered probable when muscle strength is decreased. The observed decline in muscle strength is a leading diagnostic feature. The diagnosis might be confirmed by low muscle quantity or quality [6]. Sarcopenia was also defined by the Asian Working Group for Sarcopenia (AWGS), which focused on age-related muscle and physical performance decline and created a similar algorithm for diagnosis [9]. However, in the real world sarcopenia in PCa patients is underestimated.

It should be highlighted that ADT might also influence muscle health. An association between ADT and a decrease in physical performance [10,11,12,13], muscle strength [11,12,13,14,15] and mass [15,16,17] was confirmed. These results might be indicative of possible sarcopenia development during treatment. The mechanism of ADT influence on muscle health is yet to be determined. In an international survey, physicians reported that 38% of patients with non-metastatic prostate cancer received ADT [12]. Therefore, a wide range of indications for treatment with ADT and its use in clinical settings require a thorough examination of the muscle-related side effects [18].

From the clinical perspective it is crucial to establish the direct link between ADT and the development of sarcopenia in men suffering from PCa. These findings might be relevant for the prevention of sarcopenia and indicate the choice of treatment options in future patients’ guidance. Therefore, the aim of this study was to assess the influence of ADT on sarcopenia development in PCa patients.

## 2. Materials and Methods

### 2.1. Search Strategies

The search was conducted through four databases: PubMed, Embase, Web of Science and Scopus. For the following search strategy, Mesh terms, Emtree terms and specific keywords were applied and adapted for each database. No filters were used.

*#1 sarcopenia OR Sarcopenias OR composition, body OR body composition OR Body Compositions OR Compositions, Body OR muscle mass OR muscle volume OR muscle quality OR skeletal muscle loss OR muscle loss*

*#2 Ca prostate OR cancer in the prostate OR cancer of the prostate OR cancer, prostate OR carcinogenesis of the prostate OR malignancies of the prostate OR malignancy of the prostate OR malignant neoplasm of the prostate OR malignant prostate tumor OR malignant prostate tumour OR malignant prostatic tumor OR malignant prostatic tumour OR malignant tumor of the prostate OR prostate cancerogenesis OR prostate carcinogenesis OR prostate gland cancer OR prostate malignancies OR prostate malignancy OR prostate malignant neoplasm OR prostate malignant tumor OR prostate malignant tumour OR prostatic cancer OR prostatic cancerogenesis OR prostatic carcinogenesis OR prostatic malignancies OR prostatic malignancy OR prostate cancer OR Prostatic Neoplasms OR Prostate Neoplasms OR Neoplasms, Prostate OR Neoplasm, Prostate OR Prostate Neoplasm OR Neoplasms, Prostatic OR Neoplasm, Prostatic OR Prostatic Neoplasm OR Cancers, Prostate OR Prostate Cancers OR Cancer, Prostatic OR Cancers, Prostatic OR Prostatic Cancers OR Cancer of Prostate*

*#3 Antagonists, Androgen OR Antiandrogens OR Androgen Antagonist OR Antagonist, Androgen OR Antiandrogen OR Anti-Androgen Effect OR Anti Androgen Effect OR Effect, Anti-Androgen OR Antiandrogen Effect OR Effect, Antiandrogen OR Antiandrogen Effects OR Effects, Antiandrogen OR Anti-Androgen Effects OR Anti Androgen Effects OR Effects, Anti-Androgen OR Androgen Antagonists OR ADT OR androgen deprivation therapy OR androgen suppresion therapy OR anti androgen OR antiandrogen agent OR antiandrogenic agent OR antiandrogenic drug OR nonsteroidal anti androgen OR nonsteroidal anti androgens OR nonsteroidal anti-androgen OR nonsteroidal anti-androgens OR nonsteroidal antiandrogen OR nonsteroidal antiandrogens OR gonadotropin-releasing hormone antagonist OR gonadotropin-releasing hormone antagonists OR gonadotropin-releasing hormone agonist OR gonadotropin-releasing hormone agonists OR LHRH agonist OR LHRH antagonist OR MAB OR maximum androgen blockade OR CAB OR complete androgen blockade OR gonadorelin antagonist OR antigonadorelin OR gonadorelin, anti OR gonadorelin antagonist OR gonadorelin agonist OR gonadorelin agonist OR gnrh antagonist OR gonadotropin releasing factor antagonist OR gonadotropin releasing hormone antagonist OR gonadotropin releasing hormone antagonists OR lh rh antagonist OR lrf antagonist OR luliberin antagonist OR luteinising hormone releasing hormone antagonist OR luteinizing hormone releasing hormone antagonist OR gnrh agonist OR gonadotropin releasing hormone agonist OR luteinising hormone releasing hormone agonist OR luteinizing hormone releasing hormone agonist OR orchectomy OR orcheotomy OR testectomy OR testis removal OR orchiectomy OR Orchidectomy OR Orchiectomies OR Orchidectomies OR Castration, Male OR Castrations, Male OR Male Castration OR Male Castrations*

*#1 AND #2 AND #3*

### 2.2. Study Selection

**Scheme 1:**
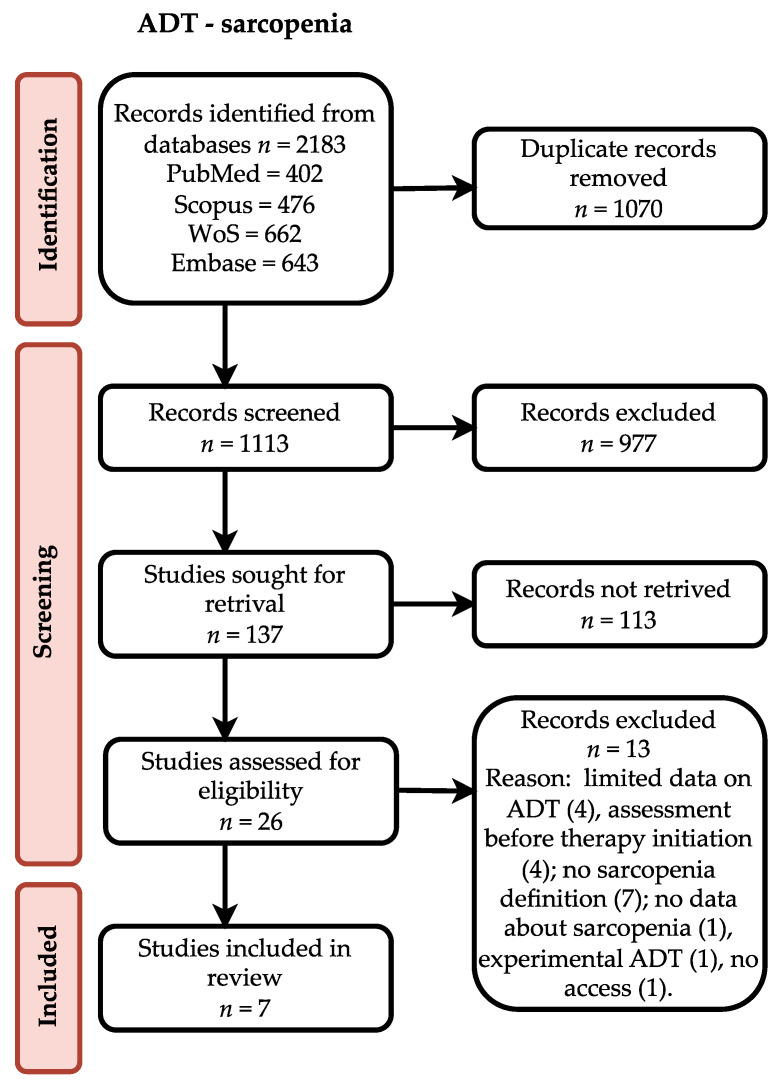
Flow diagram describing study selection.

The results of the conducted search are presented as a flow diagram according to the PRISMA statement (Scheme 1).

Each database was searched and results were analyzed with help of Zotero software (Corporation for Digital Scholarship, Vienna, Virginia, USA). First, all duplicates were removed. Then, the results were screened by two researchers. Titles were assessed and abstracts of articles possibly related to the topic were analyzed. Results in English or German were considered. Case reports, case series and conference abstracts were excluded during this phase. Occurring doubts were an indication for full-text assessment. After full-text review, the chosen studies were assessed for eligibility. For inclusion, the required criteria were sarcopenia definition, a description of the assessment method and data about ADT. Authors of reviewed articles were contacted by e-mail when necessary.

### 2.3. Data Extraction and Analysis

Studies included in the analysis were screened for the description of cancer and its treatment with a focus on ADT, clinical characteristics of patients, body composition, physical performance and details of sarcopenia measurement and prevalence. All retrieved details were double checked for mistakes and presented as tables. Collected data were assessed and eligibility for meta-analysis was discussed among researchers.

Meta-analysis was performed with the use of R Project for Statistical Computing [19]. Data in studies considered for meta-analysis were collected at baseline before ADT and at the follow up and presented in different formats. Median and quartiles were recalculated to mean and standard deviation using the package “estmeansd: Estimating the Sample Mean and Standard Deviation from Commonly Reported Quantiles in Meta-Analysis” [20]. The Method for Unknown Non-Normal Distributions (MLN) was chosen for recalculation. In the case of a change in the investigated parameters, the calculated mean was added to the mean of control and the variance of follow up was calculated as a sum of control variance and the change in parameter variance. The meta-analysis was conducted with “dmetar” package [21]. Parameters considered for assessment were BMI (Body Mass Index), SMD (Skeletal Muscle Density), SMI (Skeletal Muscle Index), SATI (Subcutaneous Adipose Tissue Index), and VATI (Visceral Adipose Tissue Index). The fixed-effect or random-effect model was chosen. Korczak et al. [22] and Sheean et al. [23] presented data for two different populations independently (patients with hormone-sensitive prostate cancer (HSPC) or castration-resistant prostate cancer (CRPC) and Black or non-Black patients, respectively). Therefore, each population was considered separately in the meta-analysis. The results of the meta-analysis were presented as a forest plot illustrating the results of the individual studies and the summary effect. Regarding details on the meta-analysis inverse variance method, the restricted maximum-likelihood estimator for tau 2 and the Q-Profile method for a confidence interval of tau 2 and tau were applied.

The heterogeneity of the studies measured with I^2^ was tested for significance [24]. Since I^2^ was found to be 0,0% for all analyzed parameters, a random-effect model was rejected and not evaluated. Egger’s Regression Test was used for the detection of potential publication bias. Importantly, a small number of publications (less than 10) made it very weak. Therefore, results indicating no bias should be treated as approximates.

### 2.4. Risk of Bias

The quality of the studies was assessed independently with the use of the Newcastle–Ottawa Quality Assessment Scale. A full score using this scale is 10 points. A study with ≥6 points was regarded as a good-quality study and each of the included studies fulfilled these criteria.

## 3. Results

The description of included studies is presented in Table 1. Observational studies were conducted in ethnically diverse groups of PCa patients, where the sarcopenia was assessed either twice (at the baseline and in the follow up) [22,23,25,26,27] or only once during the ADT treatment [28,29]. The largest group of PCa patients was found in Chiang et al. [25] and therefore this study will have the greatest impact on the meta-analysis performed. Only one qualified paper [28] had a control group of healthy individuals.

**Figure 1:**
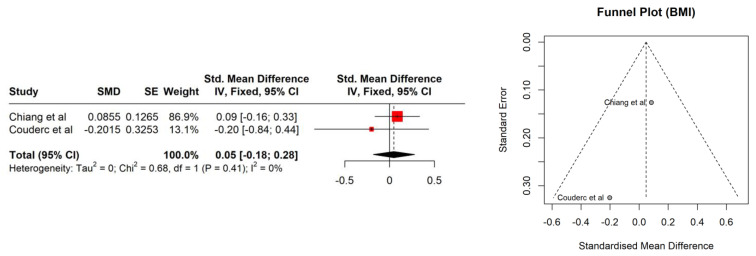
Forest plot (**left**) and funnel plot (**right**) meta-analyses of BMI.

**Figure 2:**
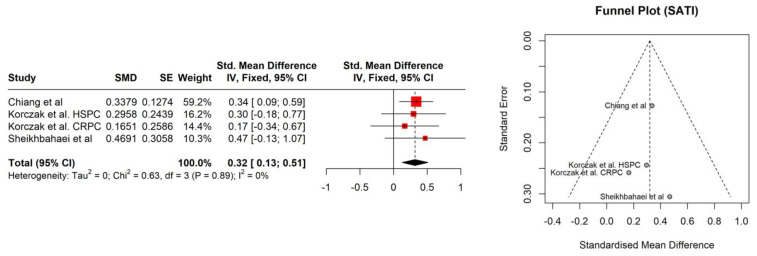
Forest plot (**left**) and funnel plot (**right**) meta-analyses of SATI.

**Figure 3:**
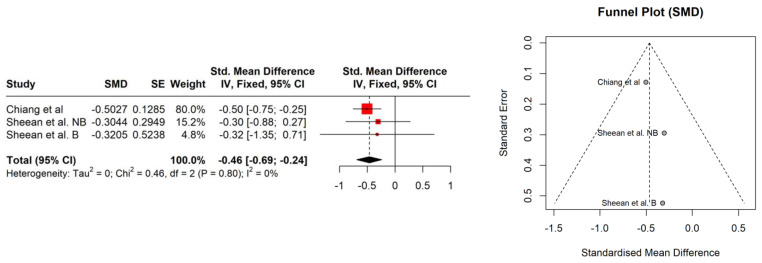
Forest plot (**left**) funnel plot (**right**) meta-analyses of SMD.

**Figure 4:**
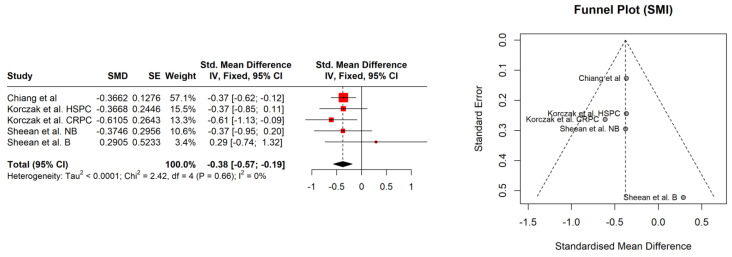
Forest plot (**left**) funnel plot (**right**) meta-analyses of SMI.

**Figure 5:**
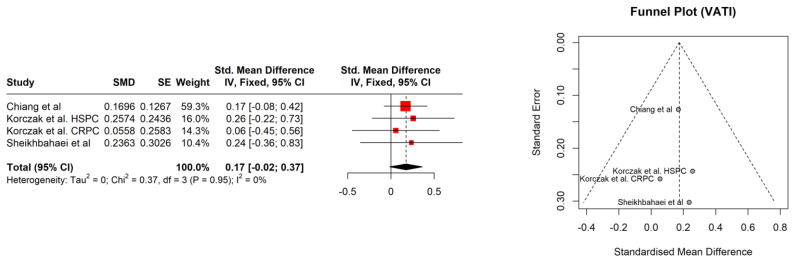
Forest plot (**left**) funnel plot (**right**) meta-analyses of VATI.

The study patients were elderly men with a number of comorbidities characterized by overweight according to Body Mass Index (BMI), with the exception of the Kimura et al. [29] study. No significant association between BMI (95% CI: −0.18–0.28, *p* = 0.686) and sarcopenia was observed (Figure 1). The hemoglobin and albumin concentrations were assessed with the normal range and the PSA level exceeded the recommended level for age. In most of the PCa patients, the median Gleason score was 8, indicating high-grade cancer (Table 2). Patients were on different treatment regimens using ADT, where the ADT duration was reported in four studies [26,27,28,29] and dietary counselling was incorporated in the treatment in only one study [22] (Table 3). Taking into account selected body composition parameters, an increase in SATI and VATI with a decrease in SMI and SMD over the course of ADT was observed (Table 4). The following parameters showed an association with sarcopenia development in PCa patients: SATI 0.32 (95% CI: 0.13–0.51) *p* = 0.001; SMD −0.46 (95% CI: −0.69 to −0.24) *p*< 0.0001; and SMI −0.38 (95% CI: −0.57 to −0.19) *p* < 0.0001 (Figure 2, Figure 3 and Figure 4). No significant association was found between sarcopenia development and VATI 0.17 (95% CI: −0.02 to 0.37), *p* = 0.074 (Figure 5). However, the sarcopenia assessment methodology and time to follow up used in single studies were differentiated, which could influence the obtained results. Nevertheless, no association with progression-free survival or overall survival was indicted [22,23,25]) (Table 5).

## 4. Discussion

The use of ADT in prostate cancer patients significantly influenced the nutritional status and therefore led to sarcopenia development. The ADT had a strong impact on the reduction in SMD (95% CI: −0.69 to −0.24) and a moderate effect on reduction in SMI (95% CI: −0.57 to −0.19) over the time period, while SATI significantly increased (95% CI: 0.13–0.51) during the treatment.

In PCa patients, during aging, we observed a decrease in the physical fitness and testosterone level when looking at the clinical settings [30]. Decreased muscle strength and size leads to the development of sarcopenia, which in extreme forms manifests itself as sarcopenic obesity associated with a significant change in body composition. ADT may accelerate unfavorable changes occurring in the patient’s body [31]. The major role is noticed on testosterone secretion that controls muscle volume and strength in men, and which is significantly decreased during ADT treatment [32]. The development of sarcopenia, understood as a decreasing amount of muscle mass or a decline in muscle quality [22], is a particular problem affecting people with chronic diseases. This applies not only to the group of men suffering from PCa and treated with ADT, but also to patients who suffer from, i.e., chronic obstructive pulmonary disease (COPD) or chronic liver disease (CLD) [33,34]. A better understanding of the regulation of muscle mass and function by androgens may have implications for PCa. Among patients with metastatic PCa, a significant effect on PFS in patients with hormone-sensitive prostate was proved [22]. The study by Korczak et al. [22] confirmed significant changes in the distribution of adipose tissue (SF, VF, SATI, and VATI) in patients who had an increase in both SF and VF, which suggests that the initiation of castration therapy in combination with docetaxel chemotherapy affects the growth of adipose tissue.

The occurrence of sarcopenia is one of the negative prognostic factors for patients with PC and therefore highlights the importance of proper diet (rich in protein and proper energy value) during the treatment to improve the effectiveness of clinical treatment. It has been proven that cancer-progression-free survival for patients with PCa and sarcopenia is shorter [35]. The current results are consistent with those obtained by other researchers [17,22,26,28]. Smith et al. [17,36] determined that taking ADT causes changes in body composition, as the muscle mass decreased with a simultaneous increase in the percentage of fat tissue. Finally, changes that occur during the treatment may cause bone fracture because bone mass density (BMD) decreases, which strongly influences the patient’s range of motion. As was highlighted by Shahinian et al. [37], patients’ sarcopenia contributes directly to a decrease in quality of life (QoL), in which physical performance is one of the main aspects that are considered.

The BMI changes were analyzed in two papers with no significant changes [25,26]. Nevertheless, it should be underlined that the BMI does not reflect real changes in the patient’s body composition, and therefore an association was not expected. Another analyzed parameter was SATI. In the current study, changes in the enlargement of subcutaneous adipose tissue were visible at a statistically significant level. The observed effect size was at the level of 0.32 (namely, statistically moderate). Current results were confirmed by Storer et al. [38], indicating that ADT causes a decrease in muscle mass and an increase in fat mass. Additionally, the SMD parameter indicated structural changes in muscle tissue. This could probably either be an increase in the water content in muscle tissue or an increase in the amount of fat in its place. Both of those mentioned factors (water and fat) have a lower HU (Hounsfield unit) than muscle tissue (less than or equal to 1000). In a study by Overkamp et al. [15], patients with PCa during ADT showed a reduction in the size of type I and II muscle fibers and their capillarization, which resulted in a reduction in SMD. In the analyzed studies, SMI showed a statistically significant decrease with an effect size of 0.38 (between moderate and large). Pablos-Rodríguez et al. [35] highlighted that this parameter is crucial in terms of survival prognosis. The decrease in SMI directly affected survival ratio [25]. As was indicated by McGovern et al. [39], this type of relation is observed not only in PCa patients, but also in others suffering from, i.e., colorectal cancer or breast cancer. The VATI parameter is recognized as an indicator for early cardiometabolic risk [40]. A study carried out by Di Bella et al. [41] confirmed that high VATI being observed in abdominal obesity has a negative effect on the disease progression in PCa patients. In the current study, the effect size for the VATI parameter was not statistically significant. Nevertheless, it is also important to assess patients’ body composition in relation to dietary habits and changes in body mass over the time period. Even the use of validated questionnaires, i.e., the NRS (Numeric Rating Scale for assessing pain) or SGA (The Subjective Global Assessment for determination a person’s nutritional status), may help to plan personalized nutrition.

Taking into account the importance of body composition in cancer patients, it seems to be justified that patients with sarcopenia should be under the care of an interdisciplinary team, including a dietitian, physiotherapist, psychologist and oncologist. Such a comprehensive approach may increase the chance of minimizing the side effects of therapy, for example, a reduction in muscle mass, worsening functioning in daily life and finally bad prognosis. There is no doubt that careful observation of the patient regarding correct eating habits and meeting energy needs is extremely important for proper treatment.

Our results showed the importance and complex consequences of sarcopenia as a part of ADT treatment. PC patients should be provided with medical, dietary, and physiotherapeutic care. Only such a comprehensive approach is able to minimize the side effects of ADT therapy, strongly influencing patients’ QoL and treatment response.

## 5. Conclusions

Androgen deprivation therapy significantly influences patients’ body composition, therefore leading to sarcopenia development. There is a need for further studies with the currently recommended methodology for sarcopenia diagnosis. Further personalized dietary interventions and proper physical activity are also required. They will have a positive influence on patients’ muscle mass. It seems that ADT with a combination of proper lifestyle interventions supplied to prostate cancer patients during the treatment may be a good strategy to reach better survival rates.

## Figures and Tables

**Table 1:** Description of included studies.

Study	Year	Country	Study Design	Study Population	Control Population	Baseline	Follow Up	ADT Implementation: Prior/Post Baseline
SARCOPENIA ASSESSED AT BASELINE AND FOLLOW-UP								
Chiang et al. [25]	2021	Italy	Retrospective observational study, single center	125	No	Before initiation of ADT+radiotherapy	Within one year after treatment initiation	Post
Korczak et al. [22]	2023	Poland	Prospective observational study, single center	HSPC 34 CRPC 30	No	HSCPC: before initiation of ADT+docetaxel±radiotherapy CRPC: during ADT+abiraterone or enzalutamide±radiotherapy±prostatectomy	At the moment of biochemical progression	HSPC post CRPC prior+post; no information about prior treatment
Sheean et al. [23]	2022	USA	Retrospective observational study	74 Subgroup at baseline: 54 Subgroup at follow-up: 19	No	Before initiation of ADT±docetaxel	At the moment of death or last contact	Post
Couderc et al. [26]	2020	France	Prospective observational study, single center	31 Subgroup at follow-up: 6	No	Before initiation of ADT+radiotherapy	At the end of the treatment	Post
Sheikhbahaei et al. [27]	2020	USA	Retrospective observational study, single center	22	No	Before initiation of neoadjuvant therapy: ADT+docetaxel	I: 1 month after the completion of neoadjuvant therapy II: approximately 1 year after initiation of neoadjuvant therapy	Post
**SARCOPENIA ASSESSED AS A SINGLE MEASURMENT DURING ADT**								
Owen et al. [28]	2019	Australia	Cross-sectional study	70	Non-ADT prostate cancer: 52 Healthy: 70	During ADT treatment ± chemotherapy		Prior
Kimura et al. [29]	2019	Japan	Cross-sectional study	89	No	During ADT		Prior

ADT, androgen deprivation therapy; HSPC, hormone-sensitive prostate cancer; CRPC, castration-resistant prostate cancer.

**Table 2:** Patients’ characteristics (baseline) n = 802.

Study	Age [Years]	PSA [ng/mL]	Gleason Score	Albumin [g/L]	Hb [mmol/l]	Performance Status	Comorbidities	BMI [kg/m^2^]	BMI Category
SARCOPENIA ASSESSED AT BASELINE AND FOLLOW-UP									
Chiang et al. [25]	73 median, 67–78 IQR	27.8 median, 21.2–60.5 IQR	N/A	N/A	N/A	N/A	Age-adjusted CCI: 4 median, 3–5 IQR	24.2 ± 3.5	N/A
Korczak et al. [22]	HSPC: 68.6 ± 7.1 CRPC: 72.0 ± 7.5	HSPC: 206.3 ± 547.6 CRPC: 82 ± 155.5	HSPC: 8 median, 5–9 rangeCRPC: 7 median, 4–9 range	N/A	HSPC: 8.176 ± 0.8370 CRPC: 8.778 ± 1.406	WHO HSPC: 0, 6%; 1, 70%; 2, 24%CRPC: 0, 100%	Diabetes HSPC: 26.5; CRPC: 16.6 Hypertension HSPC: 61.8; CRPC: 66.7 Ischemic heart disease HSPC: 14.7; CRPC: 46.76 Other HSPC: 44.1; CRPC: 40.0	HSPC: 28.5 ± 4.9 CRPC: 29.3 ± 4.4	HSPC: underweight 3%; normal: 20%; overweight: 35%; obesity: 42% CRPC: underweight 0%; normal: 19%; overweight: 37%; obesity: 44%
Sheean et al. [23]	71 median, 63–79 IQR	N = 51 ^1^ 110 median, IQR 35–677	7, 36% 8–10, 64%	N = 70 ^1^ 36 median, 34.0–41.0 IQR g/dL	N = 71 ^1^ 7.76 median, 6.83–8.5 IQR	ECOG 0, 80% ≥1, 20%	2 median, 1–3 IQR 28% diabetes	N = 71 ^1^ 27.6 median, 24.8–31.1 IQR	N = 55 ^1^ Normal 22%, overweight 46%, obesity 32%
Couderc et al. [26]	80.5 ± 4.3, 70–88 range	N/A	N/A	41.8 ± 2.5, 37.6–47.9 range	N/A	N/A	Number of comorbidities 3.4 ± 1.6, 0–7 range ≥5 drugs 58.1%	26.6 ± 3.6, 21–42 range	N/A
Sheikhbahaei et al. [27]	58 median, 54.3–64.3 IQR, 48–75 range	14 median, 7.2–44 IQR	8 median, 8–9 IQR, 7–10 range	N/A	N/A	N/A	N/A	29.3 median, 25.1–33.4 IQR	Normal 22.7%; overweight 27.3%; obese 50%
**SARCOPENIA ASSESSED AS A SINGLE MEASURMENT DURING ADT**									
Owen et al. [28]	71 ± 6	N/A	N/A	N/A	N/A	N/A	Yes 88.6% If yes, mean 3 ± 1	28.8 ± 5.0	N/A
Kimura et al. [29]	79.8 ± 6.4	0.67 ± 1.69	≤6, 16.8% ≤7, 27.0% ≥8, 56.2%	N/A	N/A	N/A	≥3, 16.9%	23.1 ± 3.0	N/A

PSA, prostate-specific antigen; Hb, hemoglobin; IQR, interquartile range; ECOG, Eastern Cooperative Oncology Group; CCI, Charlson Comorbidity Index; HSPC, hormone-sensitive prostate cancer; CRPC, castration-resistant prostate cancer. ^1^ Data collected for limited population.

**Table 3:** Cancer and treatment description.

Study	Cancer	Metastasis Sites	Current Therapy	ADT Drug	ADT Duration	Other Drugs or Interventions
SARCOPENIA ASSESSED AT BASELINE AND FOLLOW-UP						
Chiang et al. [25]	NCCN: high-risk 72.0%; very high-risk 28.0%	N/A	ADT+EBRT; 72-76Gy Followed by 2–3 years of adjuvant ADT	N/A	N/A	N/A
Korczak et al. [22]	Metastatic HSPC and CRPC	Bones HSPC: 94%; CRPC: 70%Lymph nodes HSPC: 74%; CRPC: 53% Parenchymal organs HSPC: 3%; CRPC: 0	HSPC: ADT+docetaxel 75 mg/m^2^ 79.5%/docetaxel 50 mg/m^2^ 20.5%, with radiotherapy 11.8%, prostatectomy 0 CRPC: ADT+abiraterone +predniosne 70%/enzalutamide 30% with radiotherapy 63.3%, prostatectomy 13.3%	N/A	N/A	Dietary consultation HSPC: 20%; CRPC: 38%; ONS ^2^ for approx. 50% of them if NRS ≥ 3
Sheean et al. [23]	Metastatic HSPC	Total distant metastases 1 median, 1–2 IQR Bone metastases 76%	ADT±docetaxel 34%	Androgen receptor inhibitors; gonadotropin-releasing hormone analogues; gonadotropin-releasing hormone antagonists	N/A	N/A
Couderc et al. [26]	High-risk 45.2%; intermediate 45.2%	N/A	N = 19 ^1^ ADT+radiotherapy mean 75.6 Gy	N/A	N = 19 ^1^ 6 months 42.11% 15 months 52.63% 18 months 5.26%	N/A
Sheikhbahaei et al. [27]	Castration-naïve oligometastatic cancer	N/A	Neoadjuvant ADT+docetaxel 75/55/35 mg/m^2^+dexamethasone 2/day, beginning the day before chemotherapy, for 3 days 45,45%: concurrent abiraterone+prednisone for 1–16 weeks N = 21 ^1^ radical prostatectomy+radiation therapy to the prostatic/pelvic bed+consolidative stereotactic radiation to metastases	Luteinizing hormone-releasing hormone agonists	Neoadjuvant therapy 9 weeks median, 9–11 IQR From neoadjuvant therapy to prostatectomy 9 weeks median, 7–10 IQR N = 21 ^1^ ADT for 12 months after prostatectomy	N/A
**SARCOPENIA ASSESSED AS A SINGLE MEASURMENT DURING ADT**						
Owen et al. [28]	Localized/removed 64.3%; advanced 7.1%; unknown 28.6%	N/A	ADT±prior therapy	Goserelin 57.1% Leuprorelin 20% Goserelin+bicalutamide 7.1% Leuprorelin+bicalutamide 4.3% Triptorelin 4.3% Degarelix 2.9% Abiraterone 1.4% Degarelix+bicalutamide 1.4% Enzalutamide 1.4%	Min 12 weeks; 25 ± 36 months	Previous prostatectomy 48.6% Previous radiotherapy 68.6% Previous chemotherapy 15.7%
Kimura et al. [29]	T1 10.1%; T2 39.3%; T3/T4 50.6%	N/A	ADT±prior therapy	N/A	83.6 ± 57.6 months	Prostatectomy 6.7% Radiation 21.3%

BMI, body mass index; NCCN, National Comprehensive Cancer Network; HSPC, hormone-sensitive prostate cancer; CRPC, castration-resistant prostate cancer; ADT, androgen deprivation therapy; EBRT, external beam radiation therapy; ONS, oral nutritional support; NRS, nutritional risk score. ^1^ Data collected for limited population; ^2^ 125 mL including 18 g of protein and 300 kcal.

**Table 4:** Body composition: BMI and body composition parameters.

Study	BMI [kg/m^2^] Baseline	BMI [kg/m^2^] Follow Up	Appendicular Mass [kg/m^2^]	PMI [cm^2^/m^2^] Baseline	PMI [Cm^2^/M^2^] Follow Up	SMI [cm^2^/m^2^] Baseline	SMI [cm^2^/m^2^] Follow Up	SMD [HU] Baseline	SMD [HU] Follow Up	SATI [cm^2^/m^2^] Baseline	SATI [cm^2^/m^2^] Follow Up	VATI [cm^2^/m^2^] Baseline	VATI [cm^2^/m^2^] Follow Up	TATI [cm^2^/m^2^] Baseline	TATI [cm^2^/m^2^] Follow Up
SARCOPENIA ASSESSED AT BASELINE AND FOLLOW-UP															
Chiang et al. [25]	24.2 ± 3.5	24.5 ± 3.5	N/A	N/A	N/A	47.2 ± 7.4	44.5 ± 7.3	37.8 ± 5.6	34.9 ± 5.9	39.9 ± 13.6	44.7 ± 14.7	55.2 ± 28.0	60.1 ± 29.6	95.1 ± 37.7	104.6 ± 40.4
Korczak et al. [22]	HSPC: 28.5 ± 4.9 CRPC: 29.3 ± 4.4	N/A	N/A	N/A	N/A	HSPC: 54.01 ± 9.85 CRPC: 52.0 ± 8.311	HSPC: 50.4 ± 9.61 CRPC: 47.4 ± 6.46	N/A	N/A	HSPC: 66.47 ± 33.36 CRPC: 86.1 ± 29.7	HSPC: 78.2 ± 39.2 CRPC: 92.01 ± 40.2	HSPC: 90.40 ± 44.2CRPC: 110.2 ± 37.9	HSPC: 101.7 ± 42.6 CRPC: 112.4 ± 39.89	N/A	N/A
Sheean et al. [23]	N = 71 ^1^ 27.6 median, 24.8–31.1 IQR	N/A	N/A	N/A	N/A	N = 55 ^1^ 49.8 median, 40.2–56.2 IQR	Non-Black N = 14 ^1^: −3.7 median, (−8.5)–(−0.4) IQR (7% loss) Black N = 5 ^1^: −1.2 median, (−1.8)–12.5) IQR (2% gain)	N = 55 ^1^ 33.8, median 26.3–41.8 IQR	Non-Black N = 14 ^1^: −5.7 median, (−9.6)−(3.2) IQR (14%) Black N = 5 ^1^: −5.3 median, (−7.2)−(−1.4) IQR (12%)	N = 55 ^1^ 51.9 median, 33.3–69.4 IQR	Subcutaneous adipose tissue change [cm^2^] Non-Black N = 14 ^1^: 23.2 median, (−6.5)−51.5 IQR (5% gain) Black N = 5 ^1^: 95.2 median, (89.4–172.3) IQR (8% gain)	N = 55 ^1^ 61.6 median, 33.6–98.9 IQR	Visceral adipose tissue change [cm^2^] Non-Black N = 14 ^1^: −16.0 median, (−32.2)−3.5 IQR (1% gain) Black N = 5 ^1^: median 40.1, 29.4–41.0 IQR (2% gain)	N/A	N/A
Couderc et al. [26]	N = 19 ^1^ 26.1 ± 2.6, 21–32 range	N = 19 ^1^ 25.5 ± 3.2, 18–31.9 range	ASSM/height^2^ < 7.0 for 2 patients	N/A	N/A	N/A	N/A	N/A	N/A	N/A	N/A	N/A	N/A	N/A	N/A
Sheikhbahaei et al. [27]	29.3 median, 25.1–33.4 IQR	N/A	N/A	8.7 median, 8.11–11.3 IQR Psoas muscle mass 27.5 median, 24.6–36.7 IQR	Psoas muscle areaI: −13.9% (−7.6, −16.5)II: −13.2(−6, −11.2))	N/A	N/A	N/A	N/A	72.4 median, 51.0–107.1 IQR Subcutaneous adiposity [cm^2^]: 217 median, 159.8–340.9 IQR	Subcutaneous fat [cm^2^] I: +8.9(+5.1, +21.5) II: +18.9(+6.1, +33.8)	81.6 median, 46.4–102.9 IQR Visceral adiposity [cm^2^]: 261.3 median, 142.4–326.9 IQR	Visceral fat [cm^2^] I: −1.6(−9.9, +18.7) II: +8.4(−1.7, +23.8)	Total adiposity [cm^2^] 476.8 median, 389.8–685 IQR	Total adiposity [cm^2^] I: +5.7(+0.8, +15.2)II: +10.3(+2.7, +28.5)
**SARCOPENIA ASSESSED AS A SINGLE MEASURMENT DURING ADT**															
Owen et al. [28]	28.8 ± 5.0		ALMI 8.07 ± 0.95 ALM_BMI_ 0.875 ± 0.117	N/A		N/A		N/A		N/A		N/A		N/A	
Kimura et al. [29]	23.1 ± 3.0		N/A	N/A		7.23 ± 1.10 [kg/m^2^]		0.20 ± 0.04		N/A		N/A		N/A	

BMI, body mass index; HSPC, hormone-sensitive prostate cancer; CRPC, castration-resistant prostate cancer; HU, Hounsfield units; PMI, psoas muscle index; ALM_BMI_, appendicular lean mass adjusted for body mass index; ALMI, appendicular lean mass index; ASMM, Appendicular Skeletal Muscle Mass; SMD, skeletal muscle radiodensity; SMI, skeletal muscle index; SATI, subcutaneous adipose tissue index; TATI, total adipose tissue index; VATI, visceral adipose tissue index; FFMI, fat-free mass index. ^1^ Data collected for limited population.

**Table 5:** Sarcopenia characteristics.

Study	Sarcopenia Definition	Assessment	Assessment Methodology	Sarcopenia at Baseline	Sarcopenia at Follow Up	Time to Follow Up	Significance of Outcomes	Correlation Between Survival and Sarcopenia
SARCOPENIA ASSESSED AT BASELINE AND FOLLOW-UP								
Chiang et al. [25]	SMI: <43.2 cm^2^/m^2^	CT: L3 level	One blinded researcher	33.6%	48.0%	180 days median, 146–223 IQR	*p* = 0.001	Sarcopenia at baseline was not associated with 3-year non-cancer-specific survival and 3-year prostate cancer-specific survival
Korczak et al. [22]	SMI: 1. 53 cm^2^/m^2^ 2. 43 cm^2^/m^2^ for BMI < 25 kg/m^2^ 3. Sarcopenic obesity: <53 cm^2^/m^2^ for BMI > 30 kg/m^2^	CT: L3 level	Software CoreSlicer.com. Manual correction	HSPC: 47.06% CRPC: 46.67%	HSPC: 58% CRPC: 79.3%	12.33 months median, 5.4–26 range	HSPC: NS CRPC: *p* = 0.015	Sarcopenia was not correlated with progression-free survival
Sheean et al. [23]	SMI: 1. 53 cm^2^/m^2^ 2. 43 cm^2^/m^2^ for BMI < 25 kg/m^2^ 3. Sarcopenic obesity: <53 cm^2^/m^2^ for BMI > 30 kg/m^2^	CT: L3 level	Automated tissue demarcation. Manual correction. 10 images were assessed for quality assurance	N = 55 49% (15% of N = 55 had sarcopenic obesity)	N = 8 0%	12.5 months median	N/A	Sarcopenia was not correlated with overall survival
Couderc et al. [26]	EWGSOP2 guidelines Hand-grip < 27 kg DXA ASSM/height^2^ < 7.0 kg/m^2^	Baseline: hand-grip strengthFollow-up: DXA	Hand-grip strength test was performed according to ASHT recommendations	N = 31 ^1^ Probable sarcopenia 25%	N = 6 ^1^ 1 patient	N/A	N/A	N/A
Sheikhbahaei et al. [27]	PMI < 5.7 cm^2^/m^2^ Sarcopenic obesity: PMI < 5.7 cm^2^/m^2^ for BMI > 30 kg/m^2^	CT: L3–L4 level	Manual analysis of anonymized images twice at different time points. Third reviewer assessed differences	Sarcopenic obesity 9.1%	No change at both follow up CTs	First: 3 months median, 2–3 IQR Second: 12 months median, 10.5–17.5 IQR	N/A	N/A
**SARCOPENIA ASSESSED AS A SINGLE MEASURMENT**								
Owen et al. [28]	ALM_HFM_ ≤−1.816 and weak handgrip strength and/ or slow gait speed based on EWGSOP1, EWGSOP2, FNIH and IWGS	Handgrip dynamometer 4 m walk test DXA	Six tests, three for each hand, were performed for dynamometry (the highest). Dynamometer Jamar Plus Digital Three tests for gait speed were performed (the fastest) For DXA researchers were blinded. Software 12.30.008 (Lunar iDXA, GE Lunar Corp., Madison, WI, USA)	ADT group: FNIH 1.0% EWGSOP1 1.0% EGSOP2 or IWGS 0 No-ADT group: EWGSOP1 1.92% Healthy group EWGSOP1 1.43%		N/A	N/A	N/A
Kimura et al. [29]	Walking speed < 0.8 m/s and/or handgrip strength < 26 kg+ SMI < 7.0 kg/m^2^ Sarcopenic obesity: sarcopenic criteria+body fat ≥ 25%	Handgrip dynamometer 5 m walkingBIA	Two tests were performed for dynamometry (the highest) and walking test (average) Dynamometer TKK 5401. BMI system MC-780A	Sarcopenia non-obese 15.7% Sarcopenic obesity 13.5%		N/A	N/A	N/A

CT, computed tomography; BIA, bioelectrical impedance analyzers; SMI, skeletal muscle index; PMI, psoas muscle index; HSPC, hormone-sensitive prostate cancer; CRPC, castration-resistant prostate cancer; EWGSOP1, European Working Group on Sarcopenia in Older People 2010; EWGSOP2, European Working Group on Sarcopenia in Older People 2018; FNIH, Foundation for the National Institutes of Health; IWGS, International Working Group on Sarcopenia; ASHT, American Society of Hand Therapists. ^1^ Data collected for limited population.

## Data Availability

No additional data were created during the analysis.
